# The rise of home death in the COVID-19 pandemic: a population-based study of death certificate data for adults from 32 countries, 2012–2021

**DOI:** 10.1016/j.eclinm.2023.102399

**Published:** 2024-01-02

**Authors:** Sílvia Lopes, Andrea Bruno de Sousa, Mayra Delalibera, Elizabeth Namukwaya, Joachim Cohen, Barbara Gomes

**Affiliations:** a Faculty of Medicine, University of Coimbra, Coimbra, Portugal; b NOVA National School of Public Health, Public Health Research Centre, Comprehensive Health Research Center, CHRC, NOVA University Lisbon, Lisbon, Portugal; c Palliative Care Unit, Department of Medicine, Makerere College of Health Sciences, Kampala, Uganda; d End-of-Life Care Research Group, Vrije Universiteit Brussel (VUB), Brussels, Belgium; e King’s College London, Cicely Saunders Institute of Palliative Care, Policy & Rehabilitation, United Kingdom

**Keywords:** Adult, COVID-19, Death certificates, Mortality, Palliative care

## Abstract

**Background:**

During the coronavirus 2019 disease (COVID-19) pandemic, health systems had to respond to the needs of COVID-19 patients, while caring for patients with other life-threatening conditions. Pandemics, such as the COVID-19 pandemic, stir global health and mortality patterns. This is likely to include trends in dying places. In this paper, we examine trends in place of death for adults in 32 countries, comparing the initial COVID-19 pandemic years (2020–2021) with the eight years before the pandemic (2012–2019).

**Methods:**

Data on place of death for all adults (18 years and over) that died from 1 January 2012 to 31 December 2021 were requested (47 countries approached, 32 included). The classification of place of death varied widely between countries. “Home” was the most common category, the remaining category groups comprised “hospital or health institution”, “other defined”, and “ill-defined”. We analysed place of death data in an aggregate form, by sex, age group, and selected underlying causes of death (cancer, dementia, and COVID-19).

**Findings:**

The study included 100.7 million people (51.5% male, 68.0% with ≥70 years), 20.4% died from cancer and 5.8% from dementia; 30.8% of deaths took place at home. The percentage of home deaths rose from 30.1% in 2012–2013 to 30.9% in 2018–2019 and further to 32.2% in the pandemic (2020–2021). Home deaths increased during the pandemic in 23 countries. In most countries the rise was greater in women and cancer; age differences were not consistent.

**Interpretation:**

Our study shows that there was a rise in home deaths during the pandemic, but with variability across countries, sex, age, and causes of death. The sex difference observed in most countries may have several explanations, including more engagement of women in discussions about end of life care planning and hospital admission avoidance. A higher rise of home deaths among people dying of cancer may be explained by the more predictable disease trajectory compared to non-malignant conditions, as well as earlier and better integrated palliative care.

**Funding:**

This work is part of the EOLinPLACE Project, which has received funding from the 10.13039/100010663European Research Council (ERC) under the European Union’s Horizon 2020 research and innovation programme (grant agreement No 948609).

**Research in context**

**Evidence before this study**

Deciding where one is cared for towards the end of life is a sensitive clinical issue, shaped by a combination of illness-related, individual and environmental factors. The COVID-19 pandemic may have altered reality on this matter, due to the profound impact it had on societies and care provision. To direct health policy and planning of end of life care, it is critical to examine trends in place of death, comparing pandemic and pre-pandemic years. We searched MEDLINE, Embase and PsycInfo via Ovid from inception to December 31, 2022, using search terms (place∗ or location∗ or site∗ or setting∗ or context∗) adj3 (death∗ or dying or die∗) and (death certificat∗ or death registr∗ or statistic∗ or all deaths or all persons or all individuals or all population or whole population) and (novel coronavirus, novel coronavirus or 2019 nCoV or COVID-19 or Wuhan coronavirus or Wuhan pneumonia or SARS-CoV-2), without language restrictions, to identify published papers on death certificate studies capturing pandemic trends in place of death. We excluded studies focused on pediatrics or other specific population groups (e.g., a disease group or care setting) and studies that did not present data on deaths at home. We identified seven studies conducted in Brazil, Guatemala, Italy (Rome), Japan and the UK—all showing a rise in home deaths in the pandemic compared with previous years. Data from Japan and the UK showed the excess mortality at home during the pandemic was highest in cancer. We found no studies comparing country pandemic trends.

**Added value of this study**

We have produced the largest study of international time trends in place of death to date and the first showing a rise of home death in COVID-19 pandemic across countries. The fact that it was generally rare for someone with COVID-19 to die at home (8.3% across the countries included) suggests that much of the rise of home death related to patients suffering from other life-threatening conditions. According to estimates from the Lancet Commission on Palliative Care and Pain Relief, most health conditions leading to death are amenable to palliative care.

**Implications of all the available evidence**

Our findings show an increase in the number of home deaths, especially for women and people dying of cancer. Future studies are needed to ensure that palliative and end of life care resources are appropriately allocated to support this growing trend.

## Introduction

During the coronavirus 2019 disease (COVID-19) pandemic, health systems had to respond to the needs of COVID-19 patients. They also had to continue caring for patients with other life-threatening conditions, some of whom died from these conditions while others died from COVID-19 due to their increased risk profile. The global excess of all-cause mortality during the pandemic, estimated by the COVID-19 Excess Mortality Collaborators to have been of 120.3 deaths (113.1–129.3) per 100,000 of the population,^1^ increased pressure on hospitals and health institutions to accommodate more patients and sicker patients. At the same time, families pondered the pros and cons of a hospital admission, given the risk of COVID-19 infection and restrictions in visiting.^2^ Many patients died in hospital alone.^3^

Home has always been a relevant dying place globally. An umbrella review conducted in 2023 shows that in the context of a life-threatening condition, home is the most common preferred place for end of life care and death of patients and their family members, followed by hospice and palliative care facilities.^4^ This applies to patients of all ages, although the evidence is scarcer and of low quality on the preferences of minors, which are rarely reported directly.^4^ Reasons for wanting to be at home include individual reasons (the possibility to be surrounded by family and friends, more autonomy and sense of dignity, the ability to remember personal accomplishments and close one’s life) and environmental reasons (a comfortable, familiar and supportive environment, which may improve personal freedom). Yet not all prefer to die at home. Reasons for not wanting to remain at home towards the end of life include the patient’s poor clinical condition (symptom distress, imminent death) and caregivers’ burden.^4^ Other reasons are feeling fear, uncertainty, lack of control, frustration and isolation when being at home, a perception that specialised care is provided in hospital, and difficulties accessing medication and staff at critical moments. There is also potential for the home to represent painful memories and become a traumatic place for the bereaved.^4^

The reality in terms of where people die does not always align with people’s preferences and is somewhat different in higher and lower income regions. In the former, while for most of history the majority of people died at home, this norm began to change in the mid-20 Century, in parallel with the 2nd phase of the epidemiological transition. Hospitals became the locus of medicine and providers of cure from previously serious diseases. The shift from dying at home to dying in hospital (called by Ariès the “displacement of site of death”)^5^ occurred steadily over decades, amplified by urbanisation and immigration. By the late 70s and 80s, in several higher income nations, most of all deaths occurred in hospitals.^6^ From the 90s into the 21st Century, however, the scenario changed. The nearly universal hospitalisation trend was replaced by multiple realities. Some countries began to see a drop in hospital deaths and rise in home deaths—the USA in the 80s,^7^ Canada in the 90s, China and the UK in the 2000s.^8^ Others have seen a shift away from hospitals into care homes, e.g., Switzerland, Germany, and Belgium, especially in the late 90s and early 2000s. This was also the case in the Netherlands, though the shift reversed in 2015 after extensive reforms to control long-term care expenditures (with budget cuts and closure of long-term care facilities).^9^ In other countries the hospitalisation trend persisted, e.g., Greece, Portugal,^10^ Japan and Korea.

In contrast, in lower income regions most deaths usually take place at home. A study of data on place of death from 152 country-years in 49 countries from 2005 to 2019 estimated that 80% of people in low-income countries died at home, compared to 27% of people in high-income countries.^11^ The highest percentages were found in countries of sub-Saharan Africa and in South, East and Southeast of Asia. Both cultural and system-level factors, including disease stigma and long distances to health facilities, are likely to play a role.^12^

Pandemics stir health and mortality patterns everywhere. One such is the COVID-19 pandemic, declared a public health emergency of international concern on 30 January 2020 (with first case identified in Wuhan in December 2019) and with the end of its emergency phase on 5 May 2023. This is likely to include trends in dying places. Studying the impact of the pandemic on place of death matters because it may signal critical disruptions in end of life care. If so, it should inform health policy and planning. In this paper, we examine trends in place of death for adults in 32 countries, comparing the initial COVID-19 pandemic years (2020–2021) with the eight years before the pandemic (2012–2019).

## Methods

### Study design

Data on place of death from vital registration systems for all adults that died from 1 January 2012 to 31 December 2021 were requested from national statistical offices and/or health authorities in a selection of 47 countries (first requests sent in July 2022). These included all 27 European Union countries (where the work originated) and 20 additional countries chosen to cover variation in United Nations (UN) Regions^13^ and the Quality of Death and Dying Index 2021.^14^ Out of the 47 countries approached, 32 were included (reasons for exclusion are in Supplementary File 1). A maximum of 10 years of data per country were used (2012–2021); in 2 countries 2021 data were not yet available and in France years 2018–2019 and 2021 were not yet available. This resulted in 315 country-years of data. In Supplementary File 2, we describe all countries by UN Region, World Bank income region 2021, Quality of Death and Dying Index 2021 grade,^14^ and estimates of death registration completeness when known,^15^, ^16^, ^17^, ^18^ together with all the institutions that provided data. We report England and Wales, Northern Ireland, and Scotland (UK) separately because their data were provided by different institutions and for comparison with previous studies.^19^^,^^20^

The study is focused on adults as the deaths of minors present specificities that require a separate study, with implications mainly to pediatrics. We included people who were aged 18 years or older at the time of death, except in the Republic of Korea (aged 15 years or older) and in Brazil and Germany (20 years or older), due to limitations in the age information in the data provided from these countries.

We report the study according to the STROBE guidelines with the RECORD extension (Reporting of studies Conducted using Observational Routinely-collected Data).^21^

### Ethics

The study is part of the EOLinPLACE Project, which has been approved by the Ethics Committee of the University of Coimbra, Faculty of Medicine (CE-068-2022) and is registered in Research Registry (UIN 9213). Informed consent was not required, since all data (reporting to deceased) were provided anonymised.

### Classification of place of death

The classification of place of death varied widely between countries (Supplementary File 3), but “home” was the most consistent category, present in all countries except in Germany and Hungary. In Germany, each federal state has its own death certificate template, and national information only allows classification into hospital and non-hospital deaths. In Hungary, only two categories were available for the purpose of long time series analysis—hospital and non-hospital; the latter merged “dwelling” (private residence, not necessarily the home of the deceased) with “other”. The wording used to label “home” categories differed between the remaining 30 countries; in our analysis we included the following: home, home/non-institution, at home, private house/home/residence, domicile, decedent’s home, home of family members, home of friends, community, and courtyard. We grouped the remaining categories into “hospital or health institution”, “other defined” (which included care homes and hospice/palliative care facilities, among others) and “ill-defined” (which included unknown and other unspecified places). Categories were stable throughout the study time period, except in five countries, but changes involved the category “home” only in one country (Luxembourg) and partially—introduction of “home of a family member” and “home of friend”, representing ≤0.4% of deaths in applicable years (changes in Supplementary File 4).

### Statistics

We analysed place of death data in an aggregate form, by sex, age group (18–49, 50–69, 70–79, 80+ years) and selected underlying causes of death: cancer [International Classification of Diseases—10th version (ICD-10) codes: C0–97 except C91–95], dementia (F01–03, G30, G31), and COVID-19 (U07.1, U07.2). In one country (Greece), ICD-9 was used in years 2012–2013. We selected cancer and dementia because these two diseases are predicted to be the most important drivers of the global burden of serious health-related suffering in the future (cancer in terms of rising numbers of deaths, and dementia in proportional increase), according to projections to 2060 of serious health-related suffering as defined by the Lancet Commission on Palliative Care and Pain Relief.^20^ We selected COVID-19 as an important cause of death in 2020–2021.

We calculated the number and percentage of deaths by place of death, per country, sex, age group and cause of death. We report the variation in the number of cancer deaths and the percentage of dementia, for comparability with the above-mentioned global projections.^22^ Records for which sex was unknown were included in the study (0.002%). The frequency of deaths in “ill-defined places” was analysed.

We plotted time trends and compared home death percentages in the eight years preceding the pandemic (2012–2019) and the first two pandemic years (2020 and 2021). We plotted trends by year for countries individually and grouped by UN region^13^ and grade in the Quality of Death and Dying Index 2021.^14^ To assess if differences in the age distribution of deaths between the countries influenced the percentage of home deaths, we calculated age-standardised percentages of home deaths (taking the USA population deceased in the study period as the standard population due to its highest number of cases). For every country, we projected forward the 2016–2019 age-specific percentages of home deaths, applied to the age-specific distribution of deaths in 2020–2021, following methods of earlier UK projections on place of death.^23^ This resulted in the expected percentage of home deaths during pandemic years, if pre-pandemic age-specific percentages maintained. All statistical analyses were conducted using Excel and IBM SPSS software.

### Role of the funding source

This work is part of the project ‘EOLinPLACE: Choice of where we die: a classification reform to discern diversity in individual end of life pathways’, which has received funding from the European Research Council (ERC) under the European Union’s Horizon 2020 research and innovation programme (grant agreement No 948609). The funder had no role in the study design, data collection, analysis, interpretation, writing of the manuscript or the decision to submit it for publication. The paper reflects only the authors' view and the ERC is not responsible for any use that may be made of the information it contains.

## Results

From over 100.7 million people (51.5% male, 68.0% with ≥70 years) reported as deceased from 2012 to 2021 across 32 countries, 20.4% died from cancer (range per country: 10.8%–29.8%, Mexico and Slovenia, respectively) and 5.8% died from dementia (0.1%–18.0%, Bulgaria and Finland) (Table 1; values per country in Supplementary File 5). Women died at more advanced age (75.6% with ≥70 years vs. 59.7% in men). In 2020–2021, 12.4% of deaths were caused by COVID-19 (1.3%–20.5%, Finland and Mexico), and this percentage was lower in women (8.5% in included countries). During the study period, the number of cancer deaths increased in 17 out of 28 countries (0.2%–29.5%, Sweden and Cyprus) (Supplementary File 6). The percentage of deaths caused by dementia increased in 22/28 countries (0.1%–6.6%, Romania and Finland).

**Table 1:** Characteristics of the deceased.

	Number of deceased
All	100,733,693
Country	
Median (IQR)	1,058,435 (381,675–4,057,708)
Range (minimum–maximum)	32,215–28,271,439
Sex, n (%)	
Female	47,503,897 (48.5)
Male	50,385,201 (51.5)
Age group, n (%)	
18–49 years	7,931,023 (8.1)
50–69 years	23,438,948 (23.9)
70–79 years	21,266,510 (21.7)
80+ years	45,254,951 (46.2)
Selected causes of death, n (%)	
Cancer	17,235,191 (20.4)
Dementia	4,902,091 (5.8)
COVID-19	2,027,573 (12.4)

The percentages were calculated with the number of deaths in a group (e.g., female sex) and the number of all deaths.

For sex and age, the denominator included all countries except Republic of Korea (data unavailable for sex and study age groups). For cause of death, the denominator included all countries except France, Germany, Republic of Korea, and Uganda (data unavailable, uncoded or undisclosed). For COVID-19, the denominator excluded years 2012–2019 and Brazil (COVID-19 deaths not recorded in available data).

Overall, 30.8% of deaths took place at home (Table 2). This percentage was highest in Uganda (crude: 65.1%; age standardised: 67.0%) and lowest in Malta (crude: 13.1%; age-standardised: 13.8%). Considering all countries together, a home death was more frequent among men than women (31.9% vs. 30.5%, respectively). At country level, this difference was observed in 19/29 countries. Overall, dying at home was less frequent in the younger group (18–49 years; 26.7%) than in older people (31.4% in the aged 80+). At country level, the difference was observed in 15/29 countries. When the cause of death was cancer, the percentage of home deaths (37.7%) was higher than the observed for dementia (22.9%). At country level, the difference was observed in 14/27 countries. Home was a rare place of death for those dying from COVID-19 (8.3%). Per country, that percentage ranged from 2.0% (Malta) to 22.9% (Slovenia).

**Table 2:** Percentage of home deaths, per country (crude and age-standardised), sex, age group, and selected causes of death.

	Crude	Age-standardised	Female	Male	18–49 years	50–69 years	70–79 years	80+ years	Cancer	Dementia	COVID-19
All countries	30.8	31.3	30.5	31.9	26.7	32.1	31.8	31.4	37.7	22.9	8.3
Austria	26.6	26.9	24.9	28.5	30.9	27.6	24.6	26.8	24.6	27.1	5.4
Belgium	22.8	25.3	18.8	26.9	39.6	34.2	26.4	16.7	29.1	10.1	4.8
Brazil	20.3	21.7	20.1	20.5	15.0	17.7	19.8	26.4	16.1	30.5	–
Bulgaria	61.5	61.0	63.9	59.3	38.6	52.8	59.0	71.2	70.8	73.9	5.9
Croatia	31.4	31.6	30.4	32.4	32.2	32.7	29.4	31.9	33.9	28.6	3.4
Cyprus	19.1	18.6	19.8	18.4	18.1	15.9	16.7	21.2	16.6	20.2	2.8
Czechia	21.8	22.2	19.8	23.7	27.8	27.2	21.8	18.4	24.5	10.3	4.6
Denmark	24.2	25.3	21.4	26.9	33.2	30.8	25.5	20.4	26.0	10.6	6.9
Estonia	29.9	30.4	26.4	33.8	34.5	38.0	29.8	25.3	27.2	15.0	4.5
Finland	18.0	21.0	13.5	22.5	44.6	32.5	19.4	10.4	10.7	7.1	3.3
France	21.1	22.5	18.7	23.4	32.9	26.8	22.0	18.1	–	–	–
Greece	42.7	39.2	45.3	40.2	22.8	30.0	35.3	49.7	38.6	59.1	4.6
Italy	38.0	36.9	38.7	37.3	31.2	35.2	34.9	39.9	41.9	38.9	4.1
Latvia	41.8	41.8	40.7	43.0	38.4	42.9	39.4	43.0	44.3	31.7	4.2
Lithuania	33.6	33.7	32.7	34.6	33.6	35.7	31.1	33.7	30.7	33.9	3.9
Luxembourg	18.1	19.2	15.8	20.3	27.2	24.9	18.1	14.8	11.0	8.9	4.4
Malta	13.1	13.8	12.0	14.2	19.4	16.4	14.0	11.0	9.9	5.2	2.0
Mexico	46.7	51.5	49.9	44.2	28.6	39.7	48.8	64.2	59.7	68.8	14.8
Netherlands	32.7	35.4	28.2	37.5	46.0	47.8	38.6	24.2	54.5	7.1	8.8
Poland	36.5	36.4	36.5	36.5	33.4	36.2	32.8	39.0	38.8	43.4	6.8
Portugal	25.2	24.1	27.3	23.2	20.5	20.4	22.0	28.1	17.0	28.7	5.3
Republic of Korea	15.8	–	–	–	–	–	–	–	–	–	–
Romania	58.4	58.3	60.6	56.4	35.7	47.6	57.9	69.2	59.7	62.0	7.6
Slovakia	28.4	28.5	27.2	29.5	27.0	29.1	26.1	29.5	28.8	28.9	5.7
Slovenia	44.1	42.8	47.4	40.7	34.3	37.6	38.4	49.8	42.0	73.7	22.9
Spain	26.3	25.2	27.2	25.5	19.6	22.4	23.8	28.5	27.1	29.4	5.4
Sweden	18.7	22.2	15.7	21.9	39.5	31.9	22.8	12.8	24.1	3.2	4.2
Uganda	65.1	67.0	63.6	65.8	65.0	62.4	66.7	70.4	–	–	–
UK–England and Wales	24.3	26.3	21.3	27.4	35.7	33.9	28.2	19.0	33.0	10.2	5.4
UK–Northern Ireland	28.4	29.9	25.5	31.5	42.4	37.9	31.2	22.2	38.1	14.4	6.6
UK–Scotland	27.6	29.7	23.3	32.1	60.1	39.0	28.6	18.7	32.5	9.3	6.4
United States of America	31.1	31.1	29.5	32.7	31.6	35.2	32.4	28.0	44.6	24.0	7.1

The percentages were calculated with the number of home deaths and the number of all deaths.

Germany and Hungary are not presented, since their place of death data did not include a category for home death.

For Brazil, France, Republic of Korea, and Uganda empty cells are due to data unavailable or undisclosed.

To calculate age-standardised percentages of home deaths, USA population deceased in the study period was defined as the standard population.

The percentage of home deaths rose from 30.1% in 2012–2013 to 30.9% in 2018–2019 (before the pandemic) and further to 32.2% during the pandemic (2020–2021) (Table 3). When comparing the two pandemic years (2020–2021) with the previous two years (2018–2019), home deaths increased in most countries (23/29). The increase was highest in the Northern Ireland (27.5% in 2018–2019 and 33.1% in 2020–2021) and lowest in Belgium (22.5% and 22.8%). When home deaths decreased (6/29), the highest difference was observed in Uganda (76.6% to 47.8%). In the latter, there was a trend of decreasing home deaths since 2012–2013 that accentuated during the pandemic. At country level, comparing observed and expected home death percentages for 2020–2021 based on pre-pandemic values provided similar results (Table 3). Compared with the expected, the observed home death percentages were higher in 21/29 countries. The highest difference was observed in Northern Ireland and Scotland (+5.7% percentual difference in both countries) and lowest in Bulgaria (−6.6% percentual difference).

**Table 3:** Percentage of home deaths, per country, and period.

	2012–2013	2014–2015	2016–2017	2018–2019	2020–2021 (observed)	2020–2021 (expected)
All countries	30.1	30.2	30.3	30.9	32.2	31.1
Austria	26.9	27.0	26.9	26.0	26.4	26.4
Belgium	23.3	23.0	22.4	22.5	22.8	22.0
Brazil	20.8	20.4	20.4	20.2	20.0	20.2
Bulgaria	66.3	64.9	62.6	60.6	54.8	61.4
Croatia	35.2	33.0	30.7	29.0	29.5	29.8
Cyprus	15.4	17.9	17.1	19.7	24.0	18.5
Czechia	20.6	20.7	21.3	22.5	23.5	21.8
Denmark	19.2	23.2	25.2	25.6	27.4	25.2
Estonia	33.7	30.5	28.9	27.5	28.9	28.0
Finland	19.1	17.4	18.0	18.0	17.2	17.6
France	21.2	20.9	20.8	–	21.7	20.6
Greece	42.7	42.8	42.8	42.2	42.9	42.6
Italy	39.2	38.6	37.8	36.7	37.5	37.4
Latvia	45.1	42.2	41.0	39.8	40.6	40.5
Lithuania	36.9	35.3	32.6	30.2	32.9	31.4
Luxembourg	18.6	18.4	17.0	17.5	18.8	17.1
Malta	13.2	11.9	11.6	12.2	16.2	11.9
Mexico	45.1	46.2	46.6	46.4	48.1	45.7
Netherlands	30.7	32.8	33.3	32.9	33.7	32.9
Poland	37.0	35.9	35.6	35.3	38.1	35.4
Portugal	27.4	26.0	24.9	24.9	23.2	25.0
Republic of Korea	18.3	16.1	14.9	14.1	16.0	–
Romania	65.8	62.8	58.1	53.8	53.1	56.1
Slovakia	29.9	29.1	27.5	27.1	28.4	27.2
Slovenia	42.7	43.6	43.8	43.7	46.2	44.0
Spain	26.1	25.4	26.9	25.5	27.6	26.2
Sweden	16.8	17.8	18.8	19.3	20.9	18.9
Uganda	81.8	79.3	80.3	76.6	47.8	77.8
UK–England and Wales	22.3	22.9	23.6	24.1	28.1	23.9
UK–Northern Ireland	26.9	26.6	27.3	27.5	33.1	27.4
UK–Scotland	24.9	25.4	26.6	27.5	32.7	27.0
United States of America	28.8	29.8	30.8	31.7	33.6	31.4

The percentages were calculated with the number of home deaths and the number of all deaths.

Germany and Hungary are not presented, since their place of death data did not include a category for home death.

For France, the empty cell is due to data unavailable.

**Fig. 1:**
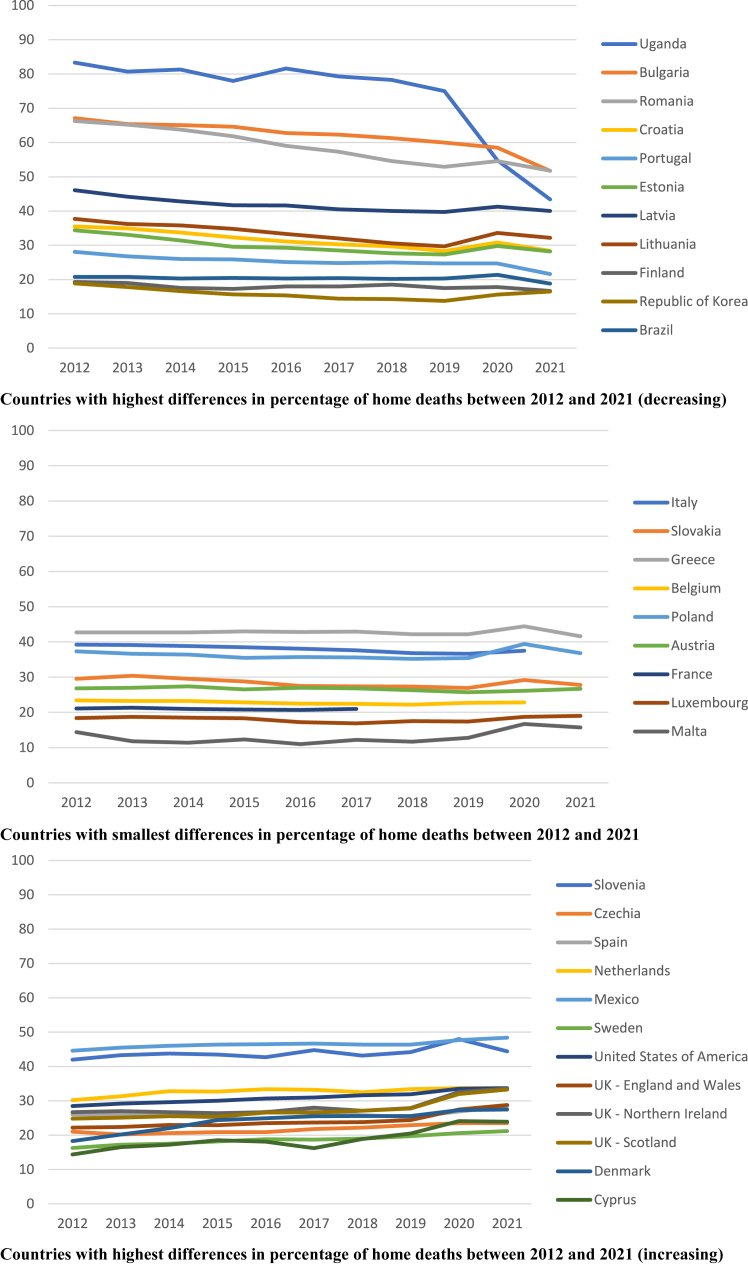
**Percentage of home deaths per country, and year.** Footnote: The figure shows home death (%) by year of death for countries grouped according to variation of the percentage of home deaths between 2012 and 2021 (or nearest year). The percentage was calculated with the number of home deaths and the number of all deaths. Germany and Hungary do not include the category home in the data provided.

Analysing year by year, we saw that the overall percentage of home deaths varied between 30.0% and 30.9% from 2012 to 2019, and then rose to 32.3% in 2020 and 32.1% in 2021 (Fig. 1 and Supplementary File 7 provide data for each country and all countries together). In 2020, the percentage of home deaths increased in all countries except Uganda (75.0% in 2019 to 54.8% in 2020) and Bulgaria (60.0% in 2019 to 58.5% in 2020). Despite the observed country variability in trends (Fig. 1), there were no apparent patterns according to UN region and Quality of Death and Dying Index grade (Supplementary Figures S1 and S2 in Supplementary File 8).

A rise of home deaths was observed for both sexes (female: 30.7%–32.2%; male: 32.2%–33.1%), when comparing 2018–2019 with 2020–2021, but was greater in women (Table 4). At country level, that pattern was observed in 17/28 countries (Supplementary File 9). The rise of home death was also observed across all age groups except in those aged 50–69 years (32.3% both in 2018–2019 and 2020–2021), but it was greater among older people (80+ years: 31.8%–34.0%). Per country, comparing the youngest and oldest age groups, the increase of home death was higher in those aged 18–49 years in 12/28 countries and in those aged 80+ years in 11/28 countries (remaining 5/28 showed home death increase in at least one group or same percentage in both). The increase of home death from 2018–2019 to 2020–2021 was seen in cancer and dementia, but was highest in cancer (cancer: 35.9%–43.0%; dementia: 22.4%–27.5%). This pattern was observed in 20/27 countries. The rise in cancer home deaths happened in 27/27 countries (range per country: 1.0%–13.0% percentual difference, Bulgaria and Northern Ireland, respectively). The rise in dementia home deaths happened in 25/27 countries (0.3%–10.2% percentual difference, Denmark and Mexico).

**Table 4:** Percentage of home deaths, per sex, age group, selected causes of death, and period.

	2012–2013	2014–2015	2016–2017	2018–2019	2020–2021
All	30.1	30.2	30.3	30.9	32.2
Sex					
Female	29.7	29.8	29.9	30.7	32.2
Male	31.2	31.4	31.6	32.2	33.1
Age group					
18–49 years	25.7	26.1	26.9	27.0	27.4
50–69 years	31.9	31.9	32.1	32.3	32.3
70–79 years	31.6	31.4	31.3	31.6	32.6
80+ years	30.1	30.4	30.6	31.8	34.0
Selected causes of death					
Cancer	36.8	36.7	36.4	35.9	43.0
Dementia	20.8	21.3	21.8	22.4	27.5
COVID-19	–	–	–	–	8.3

The percentages were calculated with the number of home deaths and the number of all deaths.

Some countries are not included due to unavailable, uncoded or undisclosed data (Germany and Hungary: category for home death; Republic of Korea: sex and study age groups; France and Uganda: cause of death).

The percentage of deaths occurring at a hospital or health institution was 47.5% (24.2%−74.5%, Netherlands and Republic of Korea) (Supplementary File 10). Deaths in this category group showed a decreasing trend from 2012–2013 (48.0%) to 2018–2019 (46.8%) and then rose to 47.6% during the pandemic. In countries where the category group “other defined places” existed, it accounted for 17.7% of deaths (0.2%–40.4%, Bulgaria and Netherlands). This category group also showed an increasing trend before the pandemic (17.9%–18.5%) and decreased in 2020–2021 (15.6%). Finally, for 11.1% of the deceased, the place where death happened was ill-defined. Where this category group was recorded, the percentage ranged from 0.3% (Luxembourg) to 54.6% (Germany, where available data at national level only allows classification into hospital and non-hospital—the latter considered ill-defined). Per period, the percentage of ill-defined places ranged between 10.8% (2016–2017) and 11.5% (2018–2019).

## Discussion

There has been a rise of home death during the pandemic years. This rise happened in four-fifths of the studied countries, regardless of prior trends. This is the largest study of international time trends in place of death to date, including 315 country-years of data on over 100 million people dying in a decade across 32 countries.

The fact that it was generally rare for someone with COVID-19 to die at home (overall 8.3%) suggests that much of the rise of home death related to patients suffering from other life-threatening conditions, most of which are amenable to palliative care.^22^ The reasons for dying at home in this context can be several and complex.^4^^,^^24^ Decisions about place of care and death during the pandemic had to be done rapidly, considering the changing status of patients, families and care systems.^25^ The pandemic may have strengthened the reasons for which people often prefer to die at home (e.g., autonomy, comfort, peace, family presence). It may also have exacerbated the reasons for preferring against some institutional places (e.g., impersonal care) and added new ones (e.g., fear of COVID-19 infection, lack of hospital beds/resources for non-COVID-19 situations). The expansion of telemedicine during the pandemic may have also played a role, allowing more patients and caregivers to receive support at short notice from a health professional while being at home.^26^ This is likely to continue in the future. On the other hand, it is possible that some patients were not able to get timely urgent care (e.g., due to stretched care resources or isolation) and therefore died at home. While qualitative research may help shed light on the reasons, our findings raise the question of whether the rising home death trend was accompanied by a reallocation of resources (human, material, financial) to ensure those dying at home and their families got the appropriate end of life care they needed, including palliative care.

The provision of palliative care at home was already challenging before the pandemic. Estimations of the coverage of specialist palliative care services across the WHO European Region in 2012 for home palliative care teams were 52% in Western European and 14% in Central and Eastern.^27^ In 2014 the WHO recognised these international challenges, and passed unanimously a landmark resolution that called for all state members to strengthen palliative care services, as a key part of their health systems.^28^ Emphasis was given to primary care, community, and home-based care. In the 2019 WHO Country Capacity Survey for the Prevention and Control of Noncommunicable Diseases, which included questions on the palliative care policy and service development in 194 member states, only 39% reported general availability (reaching at least half of patients in need) in primary health care and 40% in community- or home-based care.^29^

Evidence is building up on the services provided to people dying at home and in other places during the pandemic and their experiences.^30^^,^^31^ This may catalyse improvements in end of life care. In the meantime, our findings flag the need to monitor whether the rise of home death is kept or reverts beyond 2021, as this has implications for the allocation of healthcare resources and how services are organised to care for the dying in future years. A post-pandemic Scottish mortality study suggests that home deaths may not return to pre-pandemic levels.^32^

Our study also shows the variability in place of death between countries. This was unexplained by differences in the age composition of the populations, as age-standardised home death percentages produced similar results (Table 2). Contrasting trends were already observed pre-pandemic (Fig. 1).

The countries included in our study varied widely in Quality of Death and Dying Index grade, but we found no variation patterns by Index grade (Supplementary File 8, Supplementary Figure S1). However, it should be noted that grading information was not available for 11 countries. We also found no apparent patterns by UN region (Supplementary File 8, Supplementary Figure S2), although there was under-representation of non-European regions. A prior study with 30 European Economic Area countries has shown that there are structural economic and societal factors, along with policy choices, that influence where people die in a certain country.^33^ Other potential explanations for differences between countries may be the extent to which they have invested in national palliative care strategies that emphasise home care and death (as opposed to countries that expanded palliative care primarily in hospital), the extent of primary care reforms to strengthen collaboration with palliative care; and national reforms to control health expenditures (in particular those involving budget cuts, restrictions of stay and closure of beds in hospitals, hospice, palliative and long-term care facilities).^9^^,^^33^ In addition, differences in the type and stringiness of the restrictions that each country imposed to health and social care providers to contain the circulation of COVID-19 may have influenced the direction and magnitude of changing patterns in place of death. It is interesting to note a small decrease in the percentage of home deaths from 2020 to 2021 (32.3% and 32.1%, respectively). More frequent monitoring of mortality data may reveal finer patterns. An UK study of pandemic weekly data found home death percentages of 25% in the first wave, 33% in between the first and second wave, and 27% in the second wave.^19^ Age differences in the rise of home death were inconsistent between countries. This aligns with prior research indicating country differences in how age relates with place of death.^20^ On the other hand, in most countries, women and those dying from cancer generally showed a higher rise of home deaths. Despite being the most common patterns, these are not universal, hence variations should be considered at country level. The sex difference may have several explanations, including more engagement of women in discussions about end of life care planning and hospital admission avoidance. The influence of confounders must also be considered (women died at more advanced age and less often from COVID-19). A higher rise of home deaths among people dying of cancer may be explained by the more predictable disease trajectory compared to non-malignant conditions, as well as earlier and better integrated palliative care.^34^

Hospitals and health institutions were important places of death, both before and during the COVID-19 pandemic. The scope for COVID-19 diagnosis (and hence classification of death into this cause) was often very different between hospitals or health institutions and home, hence it is no surprise that the vast majority of deaths from COVID-19 happened in hospitals or health institutions (Supplementary File 10). Simultaneously, we observed a decrease in the percentage of “other defined places”, a category that includes, for example, hospice and palliative care facilities, nursing homes and residential aged care settings. While it appears that the rise of home deaths was due to people being diverted from these places, this interpretation warrants further research, as it may be influenced by how the categories of place of death are defined in each country, and local context. Our results also highlighted that there is considerable share of people dying in places not captured by current classifications and/or unknown. This limits the possibility of using routine data about place of death from death certificates to inform health policy and planning.

Several limitations of this study need to be discussed. Firstly, most of the countries included were high-income countries, with three upper-middle income countries (Brazil, Bulgaria and Mexico) one African low-income country (Uganda), and bias towards Europe. This flags a problem in data accessibility and limits the generalisability of the findings. An earlier international study on place of death also faced the same problem, but managed to include several of those countries to report only the overall percentage of home deaths in a given year, in some cases using subnational data.^11^ As we requested more detailed data (on all places of death by sex, age group, cause of death and several years), the data from lower income countries may be even more scarce or of limited quality if at all available. In addition, death registration completeness, ranging 90%–99.9% in the included countries except Uganda with 1.9% (see Supplementary File 2) is more compromised in lower income countries, and completeness may have changed during pandemic years although this is still unknown.^15^

Secondly, we studied only two causes of death, even if they will be the most relevant in future years.^22^ In addition, we only considered the underlying cause of death, not other contributing conditions as this information is not available in many countries.

Thirdly, our study does not consider place of care and transitions, but only the endpoint in the trajectory of end of life care.

Fourthly, the death certificate data used were not created or collected specifically for this study and the classification of place of death differs between countries. This means that categories are different and what is included in each category may also differ due to what is classified in the remaining categories. We focused on the most consistent category across countries, but still what is defined as “home” varies and limits the comparisons between countries. For example, earlier research has warned that in the USA assisted living facilities may be coded as “decedent’s home” and that in Portugal care homes may also be coded as “domicile” by the attending doctor, in absence of clear guidance for death certifiers on what places to consider in each category.^10^^,^^35^ Dying in the “community” in Uganda may include deaths taking place en route to a health facility. An international classification, which is under development as part of the EOLinPLACE Project, with a more detailed and homogeneous classification of place of death supported with instructions will enable stronger cross-country comparisons on place of death. This will also allow better mapping the actual and preferred places towards death, if used to record preferences.

Despite limitations, there are important implications from the findings. If the shift we found towards dying at home is adequately supported, aligned with preferences and associated with good outcomes, we are in the right track facing a complex health transition. If, on the other hand, deficits in end of life care are found, with the risk of failing patients and families, we must rethink and improve home support, considering reallocation of resources from other places. In the upcoming years, it is crucial to monitor whether the rise of home death is kept or reverts across countries. This will allow us to update and strengthen end of life care for all.

In conclusion, our study shows that there was a rise in home deaths during the pandemic in many countries, but with considerable within country variability. Despite home being the most preferred place of death, there is a need for evidence showing that this rising home trend was accompanied by a reallocation of resources (human, material, financial) to ensure appropriate care and support in the end of life period to those dying at home and their families.

## Contributors

SL, BG, and JC designed the study. SL, ABS, MD, and EN collected the data. SL, BG, and EN analysed the data. SL and BG verified the data. SL and BG drafted the manuscript, with contributions from JC, ABS, MD, and EN. All authors approved the version for publication.

## Data sharing statement

The data that support the findings of this study are available for some countries from the corresponding author (BG) upon reasonable request and subject to the authorisation and compliance with procedures defined by each data provider.

## Declaration of interests

BG is scientific advisor of the La Caixa Foundation Programme of Comprehensive Care for People with Advanced Diseases in Portugal since 2018.

## Appendix A Supplementary data

Supplementary Material

Abstract in Dutch

Abstract in Portuguese
