# Supplementary material for: The rise of home death in the COVID-19 pandemic: a population-based study of death certificate data for adults from 32 countries, 2012–2021
Source: eClinicalMedicine. 2024 Jan 2;68:102399. doi: 10.1016/j.eclinm.2023.102399 (PMC10965402; doi:10.1016/j.eclinm.2023.102399)
Supplement: Supplementary Material [file mmc1.pdf]

The rise of home death in the COVID-19 pandemic: a population-based study of death certificate data for adults from 32 countries, 2012-2021

Sílvia Lopes, Andrea Bruno de Sousa, Mayra Delalibera, Elizabeth Namukwaya, Joachim Cohen, Barbara Gomes

Supplementary files

Supplementary File 1. Countries approached and reasons for exclusion

Table S1. Countries approached and reasons for exclusion

	Country	Included (Y/N)	Reasons for exclusion (when applicable)
1	Angola	No	No response
2	Australia	No	Accessible data not compatible with request
3	Austria	Yes	Included
4	Belgium	Yes	Included
5	Brazil	Yes	Included
6	Bulgaria	Yes	Included
7	Burkina Faso	No	No response
8	China	No	Accessible data not compatible with request
9	Croatia	Yes	Included
10	Cuba	No	No response
11	Cyprus	Yes	Included
12	Czechia	Yes	Included
13	Denmark	Yes	Included
14	Egypt	No	Data received for years 2020-2021, not compatible with study aim
15	Estonia	Yes	Included
16	Finland	Yes	Included
17	France	Yes	Included
18	Germany	Yes	Included
19	Greece	Yes	Included
20	Hungary	Yes	Included
21	Ireland	No	Accessible data not compatible with request
22	Italy	Yes	Included
23	Latvia	Yes	Included
24	Lithuania	Yes	Included
25	Luxembourg	Yes	Included
26	Malaysia	No	Accessible data not compatible with request
27	Malta	Yes	Included
28	Marshall Islands	No	No response
29	Mexico	Yes	Included
30	Nepal	No	No response
31	Netherlands	Yes	Included
32	New Zealand	No	Data received for years 2012-2017, not compatible with study aim
33	Papua New Guinea	No	No response
34	Poland	Yes	Included
35	Portugal	Yes	Included
36	Republic of Korea	Yes	Included
37	Romania	Yes	Included
38	Samoa	No	Received a response, but data not accessible
39	Slovakia	Yes	Included
40	Slovenia	Yes	Included
41	South Africa	No	Data available online for years 2012-18, not compatible with study aim
42	Spain	Yes	Included
43	Sweden	Yes	Included

	Country	Included (Y/N)	Reasons for exclusion (when applicable)
44	Uganda	Yes	Included
45	United Kingdom	Yes	Included
46	United States of America	Yes	Included
47	Uzbekistan	No	No response

Supplementary File 2. Countries included, institutions providing the data, and country characteristics

Table S2. Countries included, institutions providing the data, and country characteristics

	Country	Institutions providing the data	UN region	World Bank income group	Quality of Death and Dying Index grade	Death registration completeness (%)
1	Austria	Statistik Austria	Western Europe	High income	n/a	98·8
2	Belgium	Statbel	Western Europe	High income	B	97·2
3	Brazil	Data were extracted from DATASUS website in February and May 2023	South America	Upper-middle income	F	97·1
4	Bulgaria	National Statistical Institute Bulgaria	Eastern Europe	Upper-middle income	n/a	99·2
5	Croatia	Croatian Bureau of Statistics	Southern Europe	High income	n/a	94·7
6	Cyprus	Ministry of Health	Western Asia	High income	n/a	97·4
7	Czechia	Czech Statistical Office	Eastern Europe	High income	F	98·3
8	Denmark	Statistics Denmark	Northern Europe	High income	C	98·9
9	Estonia	Estonian Causes of Death Registry	Northern Europe	High income	n/a	99·2
10	Finland	Statistics Finland	Northern Europe	High income	B	98·5
11	France	Epidemiology Centre on Medical Causes of Death – Inserm CépiDc	Western Europe	High income	B	97·3
12	Germany	Data were extracted from DESTATIS website in March 2023	Western Europe	High income	B	98·4
13	Greece	Hellenic Statistical Authority	Southern Europe	High income	D	94·2
14	Hungary	Hungarian Central Statistical Office	Eastern Europe	High income	B	98·2
15	Italy	Istituto Nazionale di Statistica (ISTAT)	Southern Europe	High income	n/a	96·3
16	Latvia	Centre for Disease Prevention and Control of Latvia	Northern Europe	High income	n/a	98·9
17	Lithuania	Institute of Hygiene	Northern Europe	High income	B	99·9
18	Luxembourg	Ministry of Health	Western Europe	High income	n/a	95·2
19	Malta	Ministry of Health	Southern Europe	High income	n/a	97·7
20	Mexico	National Institute of Statistics and Geography	Central America	Upper-middle income	D	90-99
21	Netherlands	Statistics Netherlands	Western Europe	High income	n/a	97·9
22	Poland	Statistics Poland	Eastern Europe	High income	B	99·0
23	Portugal	Data were received from Statistics Portugal (2012-13) and extracted from database provided by Directorate-General of Health (2014-21)	Southern Europe	High income	F	97·0
24	Republic of Korea	Statistics Korea	Eastern Asia	High income	A	98·8
25	Romania	National Institute of Public Health	Eastern Europe	High income	C	99·8
26	Slovakia	Statistical Office of the Slovak Republic	Eastern Europe	High income	D	98·5
27	Slovenia	National Institute of Public Health	Southern Europe	High income	n/a	94·7
28	Spain	National Statistics Institute	Southern Europe	High income	B	98·3
29	Sweden	Socialstyrelsen	Northern Europe	High income	B	98·1
30	Uganda	Data were extracted from database provided by National Identification & Registration Authority	Eastern Africa	Low income	C	1.9
31	United Kingdom	England and Wales: Office for National Statistics. Northern Ireland: Northern Ireland Statistics and Research Agency. Scotland: National Records of Scotland.	Northern Europe	High income	A	98·4
32	United States of America	Data were extracted from Centers for Disease Control and Prevention (CDC) website in November 2022 and May 2023	Northern America	High income	C	99·2

Footnote: n/a: not available. UN: United Nations. Sources: Quality of Death and Dying Index grades extracted from Finkelstein et al (2021)[14]. Death registration completeness estimates extracted from Adair and Lopez (2018)[15] for all countries except Mexico (WHO, 2023)[16], Republic of Korea (UN ESCAP, 2023)[17], and Uganda (NIRA, 2020)[18].

Supplementary File 3. Categories of place of death, per country

Table S3. Categories of place of death, per country

Country	Categories of place of death with recorded deaths
Austria	At home, Elderly home, Hospital, Other, Transport
Belgium	Other places / Andere plaatsen, Community (religious) / Gemeenschap (godsdienstige), Handel, bestuur / Trade, administration, Hospitaal / Hospital, Instelling / Institution, Onbekend/unkown, Openbare weg / Public road, Privéwoning / private house, Sport en ontspanning / Sport and leisure, Werkplaats (of school) / working place (or school)
Brazil	Domicílio / Home, Hospital, Outro estabelecimento de saúde/ Hospital, Other health institution, Outros/Others, Ignorado/Place unknown, Via pública/ Public road
Bulgaria	At home / Дома, Elsewhere / Другаде, Health establishment / Здравно заведение, Workplace / Месторабота
Croatia	Dwelling / stan, Medical institution / zdravstvena ustanova, Nursing home / ustanova za smještaj, Other place / drugo mjesto
Cyprus	Ambulance, Courtyard, Field, Home, Hotel, Institution, Monastery, Nursing Home, Other, Parking Place, Place unknown, Prison, Private Clinic, Public Hospital, Road, School, Sea, Swimming Pool, Town, Unspecified, Work place
Czechia	In year 2012: At home, In hospital, In medical institution, On street or public place, During transport, In welfare facility, Other<br>In years 2013-21: At home, In medical facility – inpatient care, In medical facility – other care, In the street or public place, During transport to medical facility, In social care facility, Other, Not identified
Denmark	Own home / Eget hjem, Known address / Kendt adresse, Nursing home / Plejehjem, Place with no address / Sted uden adresse, Hospital or hospice / Sygehus eller hospice, Undisclosed / Uoplyst
Estonia	In years 2012-2019 (until 30 june): Home / Kodu, Health care institution / Raviasutus, Other / Muu, Place unknown<br>In years 2019 (from 1 july) - 2021: Home / Kodu, Health care institution / Raviasutus, Other / Muu, Nursing home / Hoolekanneasutus, Workplace / Töökoht, Place unknown
Finland	2012-15: Health care unit, Private home, Abroad, Other place<br>2016-21: Health care unit, Private home, Abroad, Social care unit, Other place
France	Autres, Clinique Privee, Domicile, Hopital, Maison Retraite, Non-renseigne, Voie Publique
Germany	Hospital
Greece	Hospital / Νοσοκομείο, House / Οικία, Other place / Άλλο Μέρος, Other type of Institutional Coabitation / Άλλο Ίδρυμα Ομαδικής Συνοίκησης, Place unknown / Άγνωστο - Δεν Δηλώθηκε
Hungary	Hospital / Gyógyintézet, Other Place / Egyéb hely
Italy	Home, Public-private-accredited care institution, Hospice, Residential or socio-assistance facility, Other or unknown
Latvia	Ambulance vehicle / NMP automašīna, Home / Mājas, Hospital / Stacionārs, Military camp / base territory // Militārā nometne / bāzes teritorija, Nursing home / Pansionāts, Other places / Citur, Unknown / Nezināms
Lithuania	Home, Hospital, Other, Place unknown
Luxembourg	Airport, Ambulance, Bridge, Café / restaurant, Camping, Chocolate factory, Cinema, Day care home, Doctor's office, Farm, agricultural exploitation, Football field, Forest, Garden, Graveyard, Home, Home of family members, Home of friends, Hospital, Hotel, Lake, Maternity ward, Municipal building, National exposition hall, Other place, Place unknown, Ploughing field, Prison, Public road, Public swimming pool, Public transport, Religious convent, Retirement Home/Rehabilitation Center, River, Sports hall, Store, Technical control station for cars, Train, Vitarium (Milk factory), Walking path, Warehouse, Working place
Malta	Home, Hospital, Other, Residental Institution, Unknown
Mexico	Another place, Home, Institute for Social Security and Services for State Workers, Mexican Petroleum (Petróleos Mexicanos or Pemex), Mexican Social Security Institute, Ministry of Health (SSA), Other public unit, Place unknown, Private medical unit, Prospera - Mexican Social Security Institute, Public road, Secretariat of the Navy, Sedena - Ministry of National Defense
Netherlands	Hospital, Psychiatric institution, Nursing home, Care home, Other institutions, Home, Elsewhere, Unknown
Poland	Healthcare entity carrying out medical activities in the scope of stationary and 24-hour healthcare other than hospital, Home, Hospital, Other
Portugal	2012-13: Domicile / Num domicílio, Hospital/clinic // Em hospital/clínica, Other / Noutro local<br>2014-21: Health institution – Hospital or Local health unit/primary care unit or Other institution (these three categories are subdivided into inpatient ward, emergency department, intensive care unit, and other). Domicile, Other place, Unknown
Romania	Hospital/medical care, Home, Other

Country	Categories of place of death with recorded deaths
Republic of Korea	Medical institution, Other (including unknown location of death), Residential House, Home, Hospital/medical care, Other
Slovakia	Home / Doma, Hospital/ Nemocnica, In transit / Pri preprave, Long-stay hospital / Zariadenie pre dlhodobo chorých, Other / Iné, Street / Ulica
Slovenia	Home / Doma, Other places / Drugje, Health Institution / Zdravstvena ustanova
Spain	Domicile / Domicilio, Hospital / Hospital, Workplace or other / Lugar de trabajo u otro lugar, Missing / No consta, Nursing home / Residencia socio-sanitaria
Sweden	Hospital, Not registered, Nursing home, Other/Unknown, Private residence
Uganda	Health institution, Community
UK - England and Wales	Care home, Elsewhere, Home, Hospice, Hospital, Other communal establishment
UK - Northern Ireland	Care Home, Home, Hospice, Hospital, Other
UK - Scotland	Care Home, Home/Non-institution, Hospital, Other
United States of America	Decedent's home; Hospice facility; Hospital, Clinic or Medical Center – Dead on Arrival; Hospital, clinic or Medical Center – Inpatient; Hospital, Clinic or Medical Center – Outpatient or admitted to Emergency Room; Nursing home/long term care; Other; Place of death unknown

Supplementary File 4. Countries with changes in categories of place of death in 2012-21

Table S4. Countries with changes in categories of place of death in 2012-21

Country	Changes in categories of place of death
Czechia	Subcategorization of “medical institution” as “inpatient” or “other” and introduction of “nonidentified” place of death
Estonia	Added “nursing home” and “workplace”
Finland	Added “social care unit”.
Luxembourg	Generated every year 10-18 sub-categories that emerged from free text of the “other” category
Portugal	Subcategorization of “health institution” into primary care units (“primary care unit”, “group of health centres” or “health centre”) and “hospitals” (within: “inpatient ward”, “intensive care unit”, “emergency department” or “other”)

Supplementary File 5. Characteristics of deceased included, per country

Table S5. Characteristics of deceased included, per country

Country	Years	Total	Average per year	Female<br>n (%)	18-49 years<br>n (%)	50-69 years<br>n (%)	70-79 years<br>n (%)	80+ years<br>n (%)	Cancer<br>n (%)	Dementia<br>n (%)	COVID-19<br>n (%)
Austria	2012-2021	819,143	81,914	424,650 (51·8)	29,413 (3·6)	145,547 (17·8)	179,412 (21·9)	464,771 (56·7)	196,514 (24·0)	29,987 (3·7)	14,325 (8·0)
Belgium	2012-2020	997,567	110,841	508,015 (50·9)	39,937 (4·0)	184,495 (18·5)	198,460 (19·9)	574,675 (57·6)	233,287 (23·4)	65,588 (6·6)	22,192 (17·6)
Brazil	2012-2021	12,834,564	1,283,456	5,695,186 (44·4)	2,184,806 (17·0)	3,984,720 (31·0)	2,793,215 (21·8)	3,871,823 (30·2)	2,038,636 (15·9)	241,324 (1·9)	.
Bulgaria	2012-2021	1,133,485	113,349	543,669 (48)	56,147 (5·0)	296,290 (26·1)	308,891 (27·3)	472,157 (41·7)	173,246 (15·3)	1,619 (0·1)	36,121 (13·3)
Croatia	2012-2021	533,805	53,381	271,419 (50·8)	18,698 (3·5)	118,092 (22·1)	138,586 (26·0)	258,429 (48·4)	133,152 (24·9)	9,989 (1·9)	12,990 (10·9)
Cyprus	2012-2021	60,050	6,005	28,173 (46·9)	2,769 (4·6)	11,145 (18·6)	13,796 (23·0)	32,340 (53·9)	13,090 (21·8)	2,887 (4·8)	749 (5·4)
Czechia	2012-2021	1,142,832	114,283	560,379 (49·0)	50,656 (4·4)	274,644 (24·0)	302,480 (26·5)	515,052 (45·1)	264,491 (23·1)	32,261 (2·8)	35,569 (13·3)
Denmark	2012-2021	528,791	52,879	262,303 (49·6)	17,272 (3·3)	106,786 (20·2)	133,724 (25·3)	271,009 (51·3)	152,783 (28·9)	40,156 (7·6)	2,531 (2·3)
Estonia	2012-2021	156,452	15,645	82,042 (52·4)	9,008 (5·8)	36,897 (23·6)	36,996 (23·6)	73,551 (47·0)	36,224 (23·2)	1,782 (1·1)	1,968 (5·8)
Finland	2012-2021	532,788	53,279	267,419 (50·2)	20,551 (3·9)	103,310 (19·4)	117,213 (22·0)	291,714 (54·8)	120,676 (22·6)	95,703 (18·0)	1,500 (1·3)
France	2012-2017, 2020	4,033,586	576,227	2,017,930 (50)	176,401 (4·4)	769,449 (19·1)	686,371 (17·0)	2,401,365 (59·5)	.	.	.
Germany	2012-2021	9,259,995	926,000	4,717,291 (50·9)	279,272 (3·0)	1,675,192 (18·1)	2,151,968 (23·2)	5,153,563 (55·7)	.	.	.
Greece	2012-2021	1,221,433	122,143	598,736 (49·0)	42,282 (3·5)	194,184 (15·9)	249,013 (20·4)	735,954 (60·3)	254,643 (20·8)	17,401 (1·4)	5,028 (3·9)
Hungary	2012-2021	1,324,173	132,417	677,441 (51·2)	60,576 (4·6)	384,678 (29·1)	338,388 (25·6)	540,531 (40·8)	314,924 (23·8)	39,207 (3·0)	33,707 (11·4)
Italy	2012-2020	5,725,283	636,143	2,973,184 (51·9)	163,298 (2·9)	792,601 (13·8)	1,164,257 (20·3)	3,605,127 (63·0)	1,468,590 (25·7)	302,642 (5·3)	78,662 (10·6)
Latvia	2012-2021	288,228	28,823	152,789 (53·0)	19,290 (6·7)	72,679 (25·2)	72,441 (25·1)	123,818 (43·0)	57,421 (19·9)	4,186 (1·5)	5,045 (8·1)
Lithuania	2012-2021	412,824	41,282	210,577 (51·0)	30,134 (7·3)	103,563 (25·1)	95,434 (23·1)	183,693 (44·5)	77,729 (18·8)	4,344 (1·1)	9,282 (10·2)
Luxembourg	2012-2021	40,182	4,018	20,020 (49·8)	1,945 (4·8)	8,018 (20·0)	8,291 (20·6)	21,928 (54·6)	10,301 (25·6)	2,693 (6·7)	911 (10·4)
Malta	2012-2021	35,580	3,558	17,572 (49·4)	1,373 (3·9)	7,101 (20·0)	8,683 (24·4)	18,423 (51·8)	9,154 (25·7)	2,070 (5·8)	400 (4·9)
Mexico	2012-2021	7,147,546	714,755	311,1826 (43·5)	1,333,759 (18·7)	2,233,403 (31·2)	1,495,472 (20·9)	2,084,912 (29·2)	772,623 (10·8)	34,221 (0·5)	437,479 (20·5)
Netherlands	2012-2021	1,485,985	148,599	764,559 (51·5)	47,537 (3·2)	281,568 (18·9)	341,760 (23·0)	815,120 (54·9)	429,177 (28·9)	144,733 (9·7)	39,772 (11·9)
Poland	2012-2021	4,130,075	413,008	1,987,102 (48·1)	255,325 (6·2)	1,208,617 (29·3)	911,330 (22·1)	1,754,803 (42·5)	950,763 (23·0)	30,320 (0·7)	131,533 (13·3)
Portugal	2012-2021	1,119,303	111,930	556,384 (49·7)	44,090 (3·9)	190,163 (17·0)	233,623 (20·9)	651,427 (58·2)	262,575 (23·5)	54,959 (4·9)	20,096 (8·1)
Republic of Korea	2012-2021	2,842,261	284,226	.	.	.	.	.	.	.	.

Country	Years	Total	Average per year	Female n (%)	18-49 years n (%)	50-69 years n (%)	70-79 years n (%)	80+ years n (%)	Cancer n (%)	Dementia n (%)	COVID-19 n (%)
Romania	2012-2021	2,682,567	268,257	1,279,005 (47·7)	159,424 (5·9)	728,899 (27·2)	690,333 (25·7)	1,103,911 (41·2)	486,840 (18·1)	22,247 (0·8)	58,479 (9·3)
Slovakia	2012-2021	551,262	55,126	268,528 (48·7)	33,585 (6·1)	162,773 (29·5)	137,122 (24·9)	217,782 (39·5)	128,550 (23·3)	3,827 (0·7)	18,920 (14·4)
Slovenia	2012-2021	202,548	20,255	102,934 (50·8)	7,524 (3·7)	42,519 (21·0)	45,288 (22·4)	107,217 (52·9)	60,429 (29·8)	2,619 (1·3)	6,473 (14·0)
Spain	2012-2021	4,218,657	421,866	2,075,499 (49·2)	149,206 (3·5)	681,482 (16·2)	784,048 (18·6)	2,603,921 (61·7)	1,044,790 (24·8)	351,277 (8·3)	114,856 (12·2)
Sweden	2012-2021	912,705	91,271	464,174 (50·9)	29,182 (3·2)	135,965 (14·9)	201,739 (22·1)	545,819 (59·8)	216,607 (23·7)	89,958 (9·9)	14,752 (7·8)
Uganda	2012-2021	32,215	3,222	9,910 (30·8)	11,679 (36·3)	11,260 (35·0)	4,550 (14·1)	4,726 (14·7)	.	.	.
UK - England and Wales	2012-2021	5,322,367	532,237	2,696,997 (50·7)	233,211 (4·4)	927,741 (17·4)	1,174,633 (22·1)	2,986,782 (56·1)	1,405,794 (26·4)	618,863 (11·6)	141,066 (11·9)
UK - Northern Ireland	2012-2021	156,702	15,670	80,144 (51·1)	8,644 (5·5)	30,163 (19·2)	36,393 (23·2)	81,502 (52·0)	42,956 (27·4)	18,193 (11·6)	3,466 (9·9)
UK - Scotland	2012-2021	577,300	57,730	295,436 (51·2)	32,413 (5·6)	117,503 (20·4)	140,032 (24·3)	287,352 (49·8)	157,330 (27·3)	59,333 (10·3)	10,879 (8·6)
United States of America	2012-2021	28,271,439	2,827,144	13,782,604 (48·8)	2,381,616 (8·4)	7,417,501 (26·2)	6,072,568 (21·5)	12,399,754 (43·9)	5,721,896 (20·2)	2,577,702 (9·1)	768,822 (11·3)

Footnote: The percentages were calculated with the number of deaths in a group (e.g., female sex) and the number of all deaths. For Brazil, France, Germany, Republic of Korea, and Uganda empty cells are due to data unavailable, uncoded or undisclosed. For COVID-19, additional ICD-10 codes are used in Austria national reports.

Supplementary File 6. Evolution of the number of deceased from cancer and percentage of deceased from dementia, 2012 and 2021 or nearest

Table S6. Evolution of the number of deceased from cancer and percentage of deceased from dementia, 2012 and 2021 or nearest

	Number of deceased from cancer				Percentage of deceased from dementia		
	2012	2021 or nearest	Variation (N)	Variation (%)	2012	2021 or nearest	Variation (percentual points)
Austria	19,504	19,899	395	2·0	2·1	4·5	2·4
Belgium	26,263	25,196	-1,067	-4·1	6·2	5·8	-0·4
Brazil	179,437	222,296	42,859	23·9	1·5	1·8	0·3
Bulgaria	17,680	16,754	-926	-5·2	0·2	0·1	-0·1
Croatia	13,319	12,972	-347	-2·6	1·4	2·3	1·0
Cyprus	1,172	1,518	346	29·5	3·2	5·7	2·6
Czechia	26,416	26,114	-302	-1·1	1·9	2·9	1·0
Denmark	15,271	15,407	136	0·9	6·2	8·3	2·1
Estonia	3,497	3,556	59	1·7	0·7	1·4	0·7
Finland	11,389	12,756	1,367	12·0	14·2	20·8	6·6
Greece	27,014	28,993	1,979	7·3	0·6	2·8	2·2
Hungary	32,265	29,793	-2,472	-7·7	2·4	2·7	0·3
Italy	162,730	161,793	-937	-0·6	4·6	5·4	0·7
Latvia	5,830	5,597	-233	-4·0	0·7	1·8	1·1
Lithuania	7,722	7,503	-219	-2·8	0·5	1·5	1·0
Luxembourg	1,037	1,020	-17	-1·6	5·5	7·6	2·1
Malta	883	941	58	6·6	2·4	8·4	6·0
Mexico	68,143	84,875	16,732	24·6	0·5	0·4	-0·1
Netherlands	42,020	43,599	1,579	3·8	7·6	8·7	1·1
Poland	91,932	91,065	-867	-0·9	0·6	1·0	0·3
Portugal	24,795	26,729	1,934	7·8	1·7	6·0	4·4
Romania	47,436	45,257	-2,179	-4·6	0·6	0·7	0·1
Slovakia	11,725	12,408	683	5·8	0·5	0·4	-0·1
Slovenia	5,706	6,078	372	6·5	0·6	2·3	1·8
Spain	103,205	105,959	2,754	2·7	7·6	7·6	0·0
Sweden	21,360	21,401	41	0·2	8·6	9·8	1·2
UK - England and Wales	137,521	140,404	2,883	2·1	9·1	10·8	1·7
UK - Northern Ireland	4,016	4,474	458	11·4	10·0	11·2	1·2
UK - Scotland	15,475	16,159	684	4·4	8·7	9·9	1·3
United States of America	558,823	581,585	22,762	4·1	8·9	8·1	-0·8

Footnote: Year 2021 available for all countries except Belgium and Italy. France, Germany, Republic of Korea, and Uganda are not presented due to data unavailable, uncoded or undisclosed. For dementia deaths in Denmark, only people deceased with 70 years or more were included due to data undisclosed.

Supplementary File 7. Percentage of home deaths, per country, and year

Table S7. Percentage of home deaths, per country, and year

	2012	2013	2014	2015	2016	2017	2018	2019	2020	2021
All countries	30·0	30·1	30·2	30·1	30·2	30·3	30·9	30·9	32·3	32·1
Austria	26·8	27·0	27·4	26·5	27·0	26·8	26·3	25·7	26·1	26·7
Belgium	23·4	23·2	23·2	22·8	22·5	22·4	22·2	22·7	22·8	.
Brazil	20·8	20·8	20·3	20·5	20·3	20·4	20·2	20·3	21·4	18·8
Bulgaria	67·1	65·4	65·1	64·6	62·8	62·3	61·3	60·0	58·5	51·7
Croatia	35·5	34·9	33·7	32·3	31·1	30·3	29·7	28·3	30·8	28·4
Cyprus	14·4	16·5	17·2	18·5	18·1	16·2	18·9	20·5	24·1	23·9
Czechia	21·0	20·2	20·6	20·9	20·9	21·8	22·2	22·9	23·5	23·5
Denmark	18·3	20·2	22·0	24·4	24·9	25·5	25·6	25·6	27·3	27·5
Estonia	34·4	33·1	31·4	29·6	29·3	28·5	27·7	27·3	29·8	28·2
Finland	19·3	19·0	17·6	17·3	18·0	18·0	18·5	17·5	17·8	16·7
France	21·1	21·3	21·0	20·8	20·7	21·0	.	.	21·7	.
Greece	42·7	42·7	42·7	43·0	42·8	42·9	42·2	42·2	44·4	41·6
Italy	39·2	39·1	38·8	38·5	38·1	37·6	36·8	36·6	37·5	.
Latvia	46·1	44·2	42·8	41·7	41·6	40·5	40·0	39·7	41·3	40·0
Lithuania	37·7	36·2	35·8	34·8	33·3	32·0	30·6	29·7	33·6	32·2
Luxembourg	18·4	18·7	18·5	18·3	17·2	16·9	17·5	17·4	18·7	19·0
Malta	14·4	11·8	11·4	12·3	11·0	12·2	11·7	12·8	16·7	15·7
Mexico	44·6	45·5	46·0	46·4	46·5	46·7	46·4	46·4	47·7	48·4
Netherlands	30·2	31·3	32·8	32·7	33·4	33·2	32·5	33·4	33·7	33·7
Poland	37·3	36·6	36·4	35·5	35·7	35·6	35·2	35·4	39·4	36·8
Portugal	28·1	26·8	26·0	25·9	25·1	24·8	25·0	24·7	24·7	21·6
Republic of Korea	18·9	17·8	16·6	15·7	15·4	14·4	14·3	13·8	15·6	16·5
Romania	66·3	65·2	63·8	61·8	59·0	57·3	54·6	52·9	54·6	51·8
Slovakia	29·5	30·4	29·5	28·8	27·5	27·4	27·3	26·9	29·2	27·8
Slovenia	42·0	43·3	43·8	43·5	42·7	44·8	43·2	44·2	48·0	44·4
Spain	26·0	26·1	25·7	25·1	26·8	26·9	25·8	25·3	26·9	28·5
Sweden	16·3	17·2	17·5	18·2	18·8	18·7	19·0	19·7	20·6	21·2
Uganda	83·3	80·7	81·3	78·0	81·6	79·3	78·3	75·0	54·8	43·4
UK – England and Wales	22·2	22·4	23·0	22·9	23·5	23·7	23·8	24·4	27·5	28·8
UK – Northern Ireland	26·7	27·0	26·7	26·4	26·7	28·0	27·1	27·9	32·6	33·6
UK – Scotland	24·8	25·1	25·5	25·4	26·6	26·6	27·1	27·8	32·0	33·3
United States of America	28·5	29·2	29·6	30·0	30·7	31·0	31·6	31·9	33·5	33·7

Footnote: The percentages were calculated with the number of home deaths and the number of all deaths. Germany and Hungary are not presented since their place of death data did not include a category for home death. For Belgium, France, and Italy, empty cells are due to years unavailable.

Supplementary File 8. Trends in home deaths per country by UN region and Quality of Death and Dying Index grade

Figure S1. Trends in home deaths (%) per country by UN region

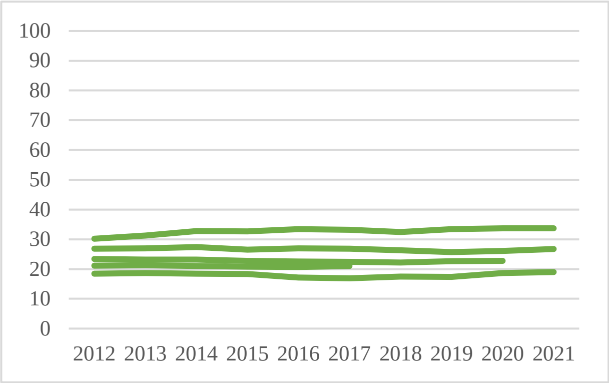

Western European countries

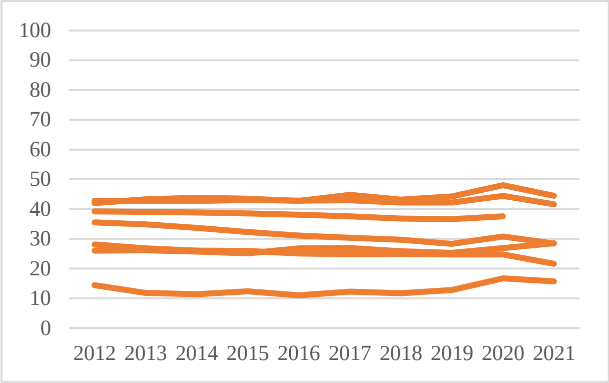

Southern European countries

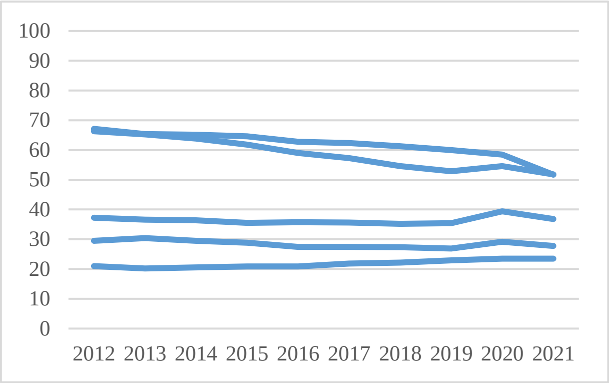

Eastern European countries

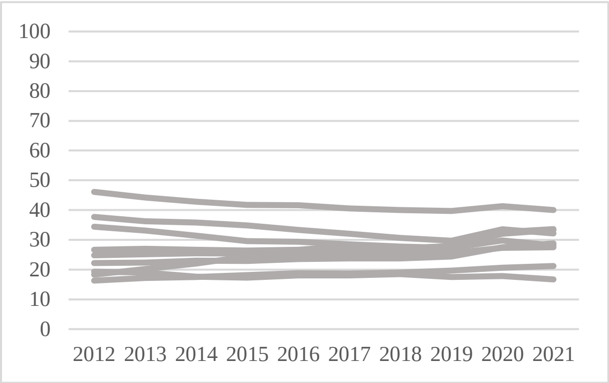

Northern European countries

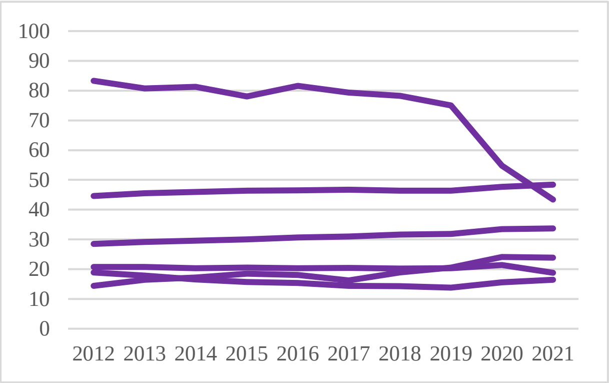

Non-European countries

Footnote: The figure shows home death (%) by year of death for countries grouped according to the UN regions.[13] The percentage was calculated with the number of home deaths and the number of all deaths. Countries by UN region: Western Europe – Austria, Belgium, France, Luxembourg, Netherlands; Southern Europe – Croatia, Greece, Italy, Malta, Portugal, Slovenia, Spain; Eastern Europe – Bulgaria, Czechia, Poland, Romania, Slovakia; Northern Europe – Denmark, Estonia, Finland, Latvia, Lithuania, Sweden, UK ((England and Wales, Northern Ireland, Scotland); non-European countries are: Brazil (South America), Cyprus (Western Asia), Mexico (Central America), Republic of Korea (Eastern Asia), Uganda (Eastern Africa), and US (Northern America). Germany and Hungary are not included in the figure as the category home is not provided in their place of death data.

Figure S2. Trends in home deaths (%) per country by Quality of Death and Dying Index grade

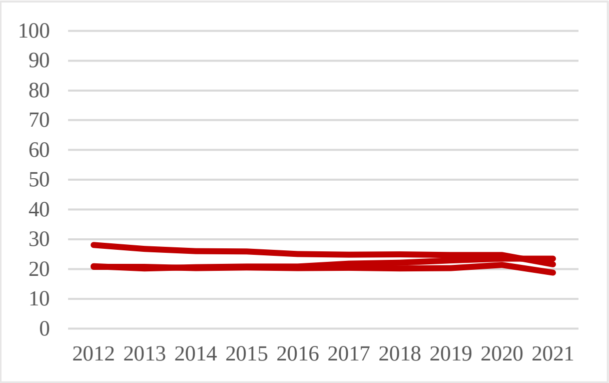

Countries with grade F (lowest)

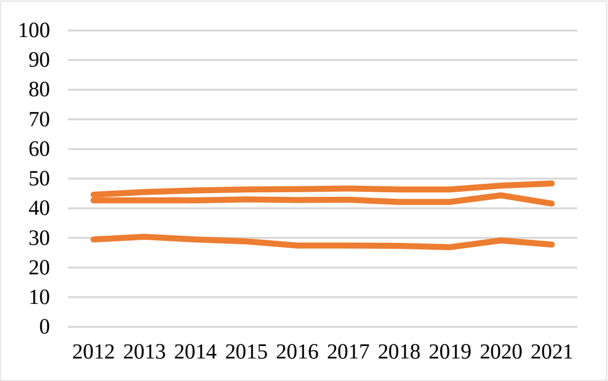

Countries with grade D

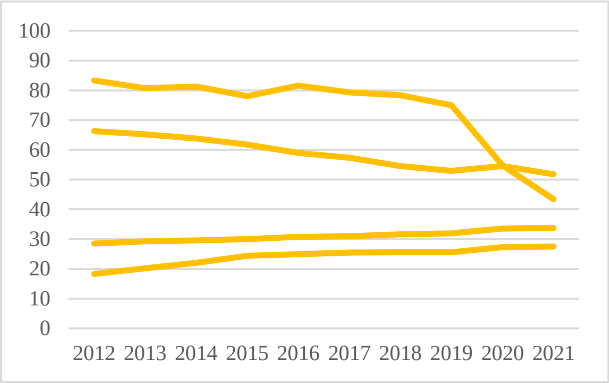

Countries with grade C

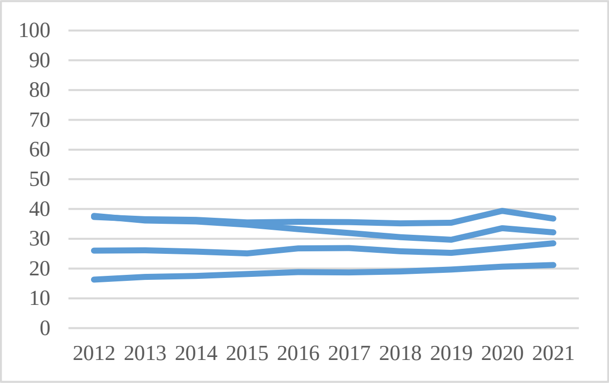

Countries with grade B

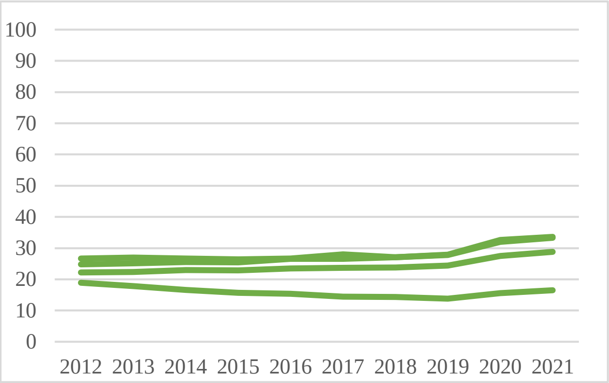

Countries with grade A (highest)

Footnote: The figure shows home death (%) by year of death for countries grouped according to the Quality of Death and Dying Index grade 2021.[14] The percentage was calculated with the number of home deaths and the number of all deaths. This ranks countries on the quality of end of life care based on assessments from country experts about 13 indicators using a preference-based algorithm. The lowest grade is F, the highest is A. Countries by grade: F) Brazil, Czechia, Portugal; D) Greece, Mexico, Slovakia; C) Denmark, Romania, Uganda, US; B) Belgium, Finland, France, Lithuania, Poland, Spain, Sweden; A) Republic of Korea, UK (England and Wales, Northern Ireland, Scotland). 13 countries are excluded from the figure: no Quality of Death and Dying Index grade available for 11 countries (Austria, Bulgaria, Croatia, Cyprus, Estonia, Italy, Latvia, Luxembourg, Malta, Netherlands, and Slovenia); Germany and Hungary do not include the category home in the data provided.

Supplementary File 9. Percentage of home deaths, per country, sex, age group, selected causes of death, and period

Table S8 – Percentage of home deaths, per country, sex, age group, selected causes of death, and period

	**Female**		**Male**		**18-49 years**		**50-69 years**		**70-79 years**		**80+ years**		**Cancer**		**Dementia**	
	2018-19	2020-21	2018-19	2020-21	2018-19	2020-21	2018-19	2020-21	2018-19	2020-21	2018-19	2020-21	2018-19	2020-21	2018-19	2020-21
All countries	30·7	32·2	32·2	33·1	26·9	27·3	32·3	32·3	31·6	32·6	31·8	34·0	35·9	43·0	22·4	27·5
Austria	24·3	24·7	27·8	28·2	31·5	32·9	28·0	29·0	23·3	23·1	26·1	26·6	23·0	25·7	24·5	26·1
Belgium	18·7	18·8	26·4	27·0	40·4	44·0	34·3	35·9	26·4	26·8	16·4	16·9	27·9	35·4	9·3	10·8
Brazil	19·7	19·8	20·7	20·1	15·8	14·8	18·1	16·6	19·4	18·9	25·1	27·3	15·2	19·3	28·5	38·6
Bulgaria	62·5	56·9	58·9	52·8	38·4	34·9	52·6	45·3	58·5	51·2	69·3	65·8	67·8	68·8	72·8	75·5
Croatia	27·4	28·4	30·7	30·7	31·3	31·6	31·7	31·1	27·3	27·3	28·4	29·9	30·4	35·3	25·2	27·6
Cyprus	20·4	25·0	19·1	23·1	20·8	21·7	17·7	22·1	17·3	20·9	21·3	26·1	16·1	23·5	18·2	25·0
Czechia	20·3	21·6	24·6	25·3	29·4	31·5	28·9	30·2	22·9	23·3	18·4	19·7	26·0	34·7	10·2	12·3
Denmark	22·7	24·6	28·3	30·0	32·3	35·3	32·3	33·8	27·0	28·5	22·0	24·3	28·2	31·6	11·9	12·2
Estonia	23·8	25·9	31·7	32·4	33·1	35·8	36·6	38·4	27·2	28·4	22·8	24·3	23·4	28·2	12·3	15·2
Finland	13·7	12·3	22·3	22·1	45·3	46·6	32·7	33·4	20·5	20·5	10·4	9·2	11·1	13·5	7·5	4·0
France	.	19·1	.	24·2	.	35·0	.	29·5	.	23·3	.	18·2	.	.	.	.
Greece	44·9	45·5	39·6	40·4	22·1	23·2	29·6	29·6	34·1	34·0	48·8	50·0	36·6	41·0	55·1	61·1
Italy	37·4	38·3	35·9	36·6	30·5	34·2	33·8	35·4	33·5	33·8	38·6	39·1	39·4	47·7	37·1	38·2
Latvia	38·5	39·8	41·3	41·5	37·3	39·1	41·5	41·6	37·2	37·8	40·7	41·7	40·4	45·0	29·5	32·5
Lithuania	28·8	32·0	31·7	33·8	32·2	33·1	33·2	35·2	28·3	30·0	29·2	33·0	24·7	33·9	30·7	36·4
Luxembourg	15·6	16·3	19·3	21·3	25·6	29·0	25·9	24·5	17·1	20·6	14·0	15·5	9·5	15·3	7·4	9·5
Malta	11·3	15·1	13·1	17·2	16·5	24·8	15·6	20·8	13·6	17·0	10·1	13·6	8·1	16·0	3·7	4·7
Mexico	49·9	51·3	43·7	45·7	27·8	30·2	39·9	40·4	48·4	50·8	63·5	68·1	57·9	65·8	66·7	76·9
Netherlands	28·4	29·8	37·7	37·5	45·0	47·2	47·5	47·4	39·2	38·8	24·8	26·4	53·6	57·8	7·6	10·0
Poland	35·1	38·7	35·5	37·5	32·9	35·0	35·4	36·7	31·8	33·3	37·3	41·9	35·4	44·8	41·2	46·3
Portugal	27·0	24·2	22·8	22·1	20·1	21·7	20·2	21·7	21·5	20·9	27·6	24·4	15·5	18·6	26·8	24·5
Romania	56·0	55·0	51·8	51·4	32·9	33·2	43·3	42·6	52·1	49·8	64·4	64·5	52·2	57·3	56·6	63·1
Slovakia	25·6	27·0	28·6	29·7	26·7	28·3	28·5	29·2	25·2	26·0	27·4	29·5	26·7	34·1	25·1	29·9

	Female		Male		18-49 years		50-69 years		70-79 years		80+ years		Cancer		Dementia	
Slovenia	47·1	50·7	40·2	41·6	32·9	33·6	36·6	39·1	37·9	38·8	49·5	52·2	40·7	49·4	67·9	76·4
Spain	25·7	28·0	25·3	27·3	26·0	29·0	23·8	26·6	23·2	24·9	26·6	28·7	24·1	32·0	26·6	31·2
Sweden	16·4	18·2	22·3	23·6	39·1	40·2	32·6	34·1	23·3	25	13·5	15·2	23·9	29·7	3·4	4·4
Uganda	76·7	42·9	76·5	50·0	77·4	46·7	72·4	48·1	79·3	48·1	81·1	49·5	.	.	.	.
UK - England and Wales	21·0	25·7	27·2	30·5	35·3	38·4	33·4	36·9	27·7	31·7	19	23·2	31·3	41·9	10·1	15·6
UK - Northern Ireland	24·9	30·7	30·2	35·5	42·2	47·9	37·2	40·8	29·4	35·7	21·7	27·6	34·7	47·7	14·4	21·6
UK - Scotland	22·9	28·5	32·2	36·8	62·7	65·3	38·5	44·3	28·1	33·8	18·7	23·8	30·3	42·2	9·3	14·2
United States of America	30·0	32·6	33·3	34·5	32·0	32·0	35·7	35·5	32·9	34	28·6	32·4	43·6	51·4	24·0	30·0

Footnote: The percentages were calculated with the number of home deaths and the number of all deaths. Some countries are not presented in the table, due to unavailable, uncoded or undisclosed data (Germany and Hungary: category for home death; Republic of Korea: sex and study age groups; France and Uganda: cause of death). For Belgium, France, and Italy, empty cells are due to years unavailable.

Supplementary File 10. Percentage of deaths in hospital, other defined places, and ill-defined places, per country, and period

Table S9. Percentage of deaths in hospital, other defined places, and ill-defined places, per country, and period

	**Hospital or health institution**	**Other defined**	**Ill-defined**
All countries	47·5	17·7	11·1
Austria	49·6	19·4	4·3
Belgium	46·8	29·0	1·4
Brazil	67·0	3·8	8·8
Bulgaria	30·5	0·2	7·8
Croatia	50·2	15·7	2·7
Cyprus	59·6	15·2	6·2
Czechia	65·2	9·9	3·2
Denmark	42·5	20·3	13·0
Estonia	58·9	3·2	8·0
Finland	62·8	11·6	7·7
France	55·5	19·7	3·6
Germany	45·4	.	54·6
Greece	49·6	5·4	2·3
Hungary	65·0	.	35·0
Italy	41·3	15	5·7
Latvia	43·5	8·5	6·2
Lithuania	58·0	.	8·3
Luxembourg	55·1	26·6	0·3
Malta	68·5	15·5	2·9
Mexico	43·0	4·1	6·2
Netherlands	24·2	40·4	2·7
Poland	58·3	.	5·3
Portugal	62·7	.	12·1
Republic of Korea	74·5	.	9·7
Romania	32·9	.	8·7
Slovakia	55·0	4·9	11·7
Slovenia	51·9	.	4·0
Spain	53·0	15·1	5·6
Sweden	38·4	35·7	7·2
Uganda	34·9	.	.
UK - England and Wales	46·3	27·3	2·0
UK - Northern Ireland	46·6	22·3	2·7
UK - Scotland	49·3	22·8	0·4
United States of America	36·4	25·9	6·5
Period			
2012-2013	48·0	17·9	11·3
2014-2015	47·7	18·5	10·9
2016-2017	47·5	18·6	10·8
2018-2019	46·8	18·5	11·5

	Hospital or health institution	Other defined	Ill-defined
2020-2021	47·6	15·6	11·2
Cause of death			
Cancer	44·2	17·1	4·3
Dementia	19·7	52·1	6·7
COVID-19	79·3	12·2	1·9

Footnote: The percentages were calculated with the number of deaths at each place (e.g., hospital or health institution) and the number of all deaths. For group of categories "other defined", calculation included only countries with that group of categories. Empty cells mean that the place of death category groups "Other defined" or "Ill-defined" were not available in the country.
