# Supplementary material for: The rise of home death in the COVID-19 pandemic: a population-based study of death certificate data for adults from 32 countries, 2012–2021
Source: eClinicalMedicine. 2024 Jan 2;68:102399. doi: 10.1016/j.eclinm.2023.102399 (PMC10965402; doi:10.1016/j.eclinm.2023.102399)
Supplement: Abstract in Dutch [file mmc2.pdf]

The rise of home death in the COVID-19 pandemic: a population-based study of death certificate data for adults from 32 countries, 2012-2021

The following translations in Dutch were submitted by the authors and we reproduce them as supplied. They have not been peer reviewed. Our editorial processes have only been applied to the original abstract in English, which should serve as reference for this manuscript.

Abstract in Dutch

Achtergrond

Tijdens de pandemie van het coronavirus in 2019 (COVID-19) moesten zorgstelsels reageren op de zorgnoden van patiënten met COVID-19, terwijl ze tegelijk zorg moesten geven aan patiënten met andere levensbedreigende aandoeningen. Pandemieën, zoals de COVID-19 pandemie, hebben een impact op mondiale gezondheids- en sterftepatronen, en waarschijnlijk ook op trends in de plaats van overlijden. In dit artikel onderzoeken we trends in de plaats van overlijden van volwassenen in 32 landen, waarbij we de initiële jaren van de COVID-19 pandemie (2020-2021) vergelijken met de acht jaar voorafgaand aan de pandemie (2012-2019).

Methoden

Gegevens over de plaats van overlijden van alle volwassenen (18 jaar en ouder) die overleden zijn tussen 1 januari 2012 en 31 december 2021 zijn opgevraagd (47 landen benaderd, 32 geïncludeerd). De classificatie van de plaats van overlijden varieerde sterk tussen landen. ‘Thuis’ was de meest voorkomende categorie, de overige categoriegroepen waren ‘ziekenhuis of zorginstelling, ‘anders gedefinieerd’ en ‘slecht gedefinieerd’. De gegevens over de plaats van

sterven zijn geanalyseerd in een geaggregeerde vorm, per geslacht, leeftijdscategorie en geselecteerde onderliggende doodsoorzaken (kanker, dementie en COVID-19).

Resultaten

In deze studie werden 100,7 miljoen mensen geïncludeerd (51,5% man, 68,0% ≥70 jaar oud), 20,4% overleed aan kanker en 5,8% aan dementie; 30,8 % van de sterfgevallen vond thuis plaats. Het percentage van sterfgevallen dat thuis plaatsvond steeg van 30,1% in 2012-13 naar 30,9% in 2018-19 en verder naar 32,2% tijdens de pandemie (2020-21). Het aantal sterfgevallen dat thuis plaatsvond is tijdens de pandemie in 23 landen toegenomen. In de meeste landen was de stijging groter bij vrouwen en bij mensen met kanker; leeftijdsverschillen waren niet consistent.

Interpretatie

Uit onze studie blijkt dat er tijdens de pandemie sprake was van een toename van het aantal sterfgevallen thuis, maar met verschillen tussen landen, geslacht, leeftijd en doodsoorzaken. Het verschil met betrekking tot geslacht, kan meerdere verklaringen hebben. Verklaringen zouden kunnen zijn dat vrouwen meer deelnemen aan gesprekken over planning van de zorg rondom het levenseinde en ziekenhuisopname vermijden. Een grotere toename in overlijdens in eigen huis onder mensen die overlijden aan kanker kan worden verklaard door het meer voorspelbare ziekteverloop in vergelijking tot niet-maligne aandoeningen, evenals eerdere en beter geïntegreerde palliatieve zorg.
