# Supplementary material for: The rise of home death in the COVID-19 pandemic: a population-based study of death certificate data for adults from 32 countries, 2012–2021
Source: eClinicalMedicine. 2024 Jan 2;68:102399. doi: 10.1016/j.eclinm.2023.102399 (PMC10965402; doi:10.1016/j.eclinm.2023.102399)
Supplement: Abstract in Portuguese [file mmc3.pdf]

The rise of home death in the COVID-19 pandemic: a population-based study of death certificate data for adults from 32 countries, 2012-2021

The following translations in Portuguese were submitted by the authors and we reproduce them as supplied. They have not been peer reviewed. Our editorial processes have only been applied to the original abstract in English, which should serve as reference for this manuscript.

Abstract in Portuguese

Contexto

Durante a pandemia da doença do coronavírus 2019 (COVID-19), os sistemas de saúde tiveram de responder às necessidades dos doentes com COVID-19 e, simultaneamente, cuidar de doentes com outras doenças potencialmente fatais. As pandemias, como a pandemia de COVID-19, alteram os padrões de saúde e mortalidade a nível mundial. É provável que tal inclua as tendências nos locais de morte. Neste artigo, analisamos as tendências no local de morte de adultos em 32 países, comparando os anos iniciais da pandemia de COVID-19 (2020-21) com os oito anos anteriores à pandemia (2012-19).

Metodologia

Foram solicitados dados sobre o local de morte para todos os adultos (18 anos ou mais) que morreram de 1 de janeiro de 2012 a 31 de dezembro de 2021 (47 países contactados, 32 incluídos). A classificação do local de morte variou consideravelmente entre países. "Casa" foi a categoria mais comum, os restantes grupos de categorias incluíram "hospital ou instituição de saúde", "outro especificado" e "não especificado". Foram analisados os dados relativos ao local de morte de forma agregada, por sexo, grupo etário e causas de morte selecionadas (cancro, demência e COVID-19).

Resultados

O estudo incluiu 100,7 milhões de pessoas (51,5% homens, 68,0% com 70 anos ou mais); 20,4% morreram de cancro e 5,8% de demência; 30,8% das mortes ocorreram em casa. A percentagem de mortes em casa aumentou de 30,1% em 2012-13 para 30,9% em 2018-19, crescendo para 32,2% na pandemia (2020-21). As mortes em casa aumentaram durante a pandemia em 23 países. Na maioria dos países, o aumento foi maior no grupo das mulheres e cancro; as diferenças por idade não foram consistentes.

Interpretação

Este estudo mostra que houve um aumento das mortes em casa durante a pandemia, mas com variabilidade por país, sexo, idade e causa de morte. A diferença por sexo observada na maioria dos países pode ter diversas explicações, incluindo maior participação das mulheres na discussão sobre planear o fim de vida e evitar o internamento hospitalar. O maior aumento de mortes em casa entre as pessoas que morreram de cancro pode ser explicado pela maior previsibilidade da trajetória da doença comparativamente a doenças não malignas, bem como por cuidados paliativos mais precoces e integrados.
